# Supplementary figures and images for: A subset of gut leukocytes has telomerase-dependent “hyper-long” telomeres and require telomerase for function in zebrafish
Source: Immun Ageing. 2022 Jul 11;19:31. doi: 10.1186/s12979-022-00287-8 (PMC9277892; doi:10.1186/s12979-022-00287-8)

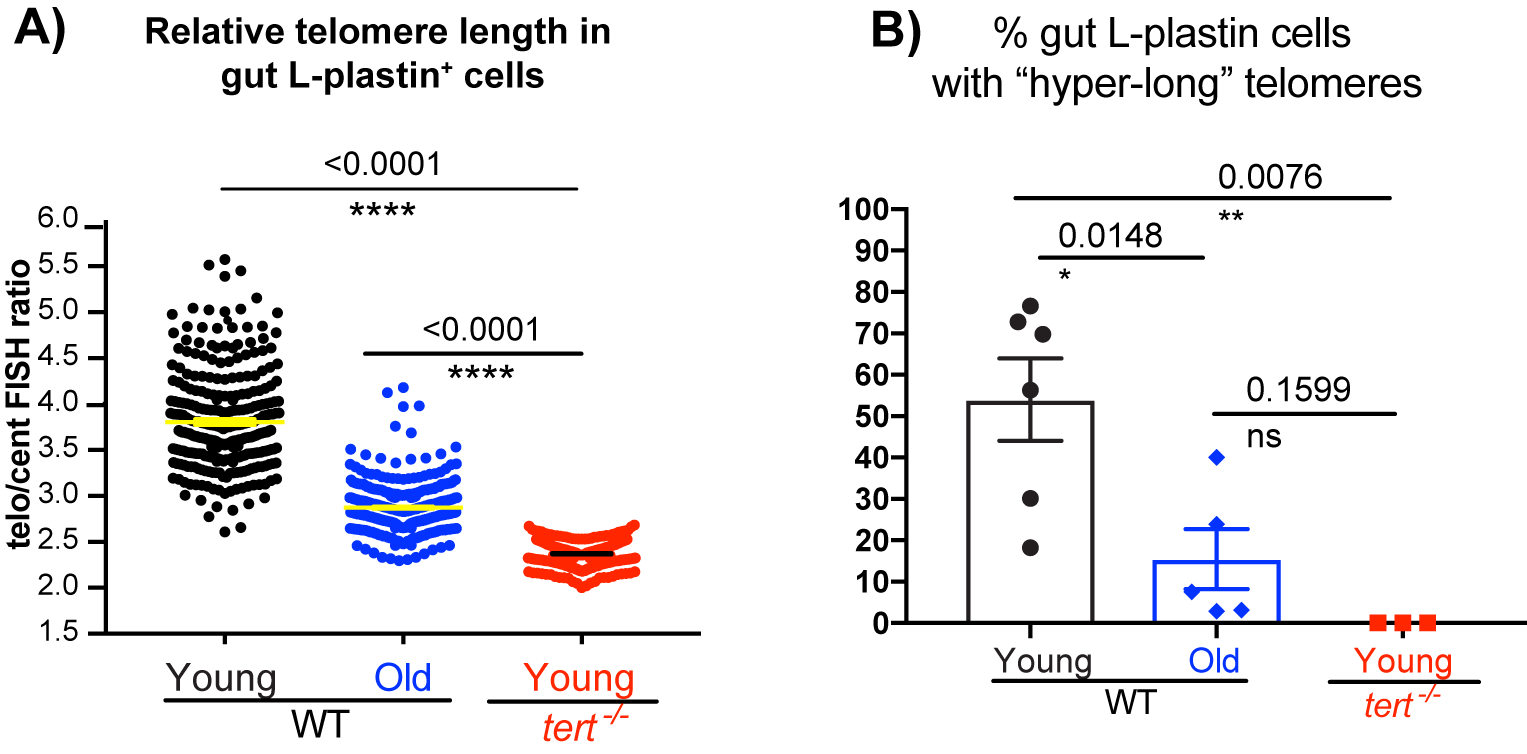

Supplement: Supplementary file 1 — Additional file 1: Supplementary Figure 1. Telomere length decreases in gut immune cells with ageing. A) Quantification of the relative telomere length of L-plastin+ cells, from gut paraffin sections, using combined immunostaining for anti-L-plastin and telomere in situ hybridization (Telo-FISH), together with the near-centromeric probe (Cent-FISH), as in Figure 1). B) From the same quantifications as in A), we can calculate the % of gut L-plastin+ cells with “hyper-long” telomeres. Young animals are c.5 months old and old animals are >30-36 months old. [file 12979_2022_287_MOESM1_ESM.tif]

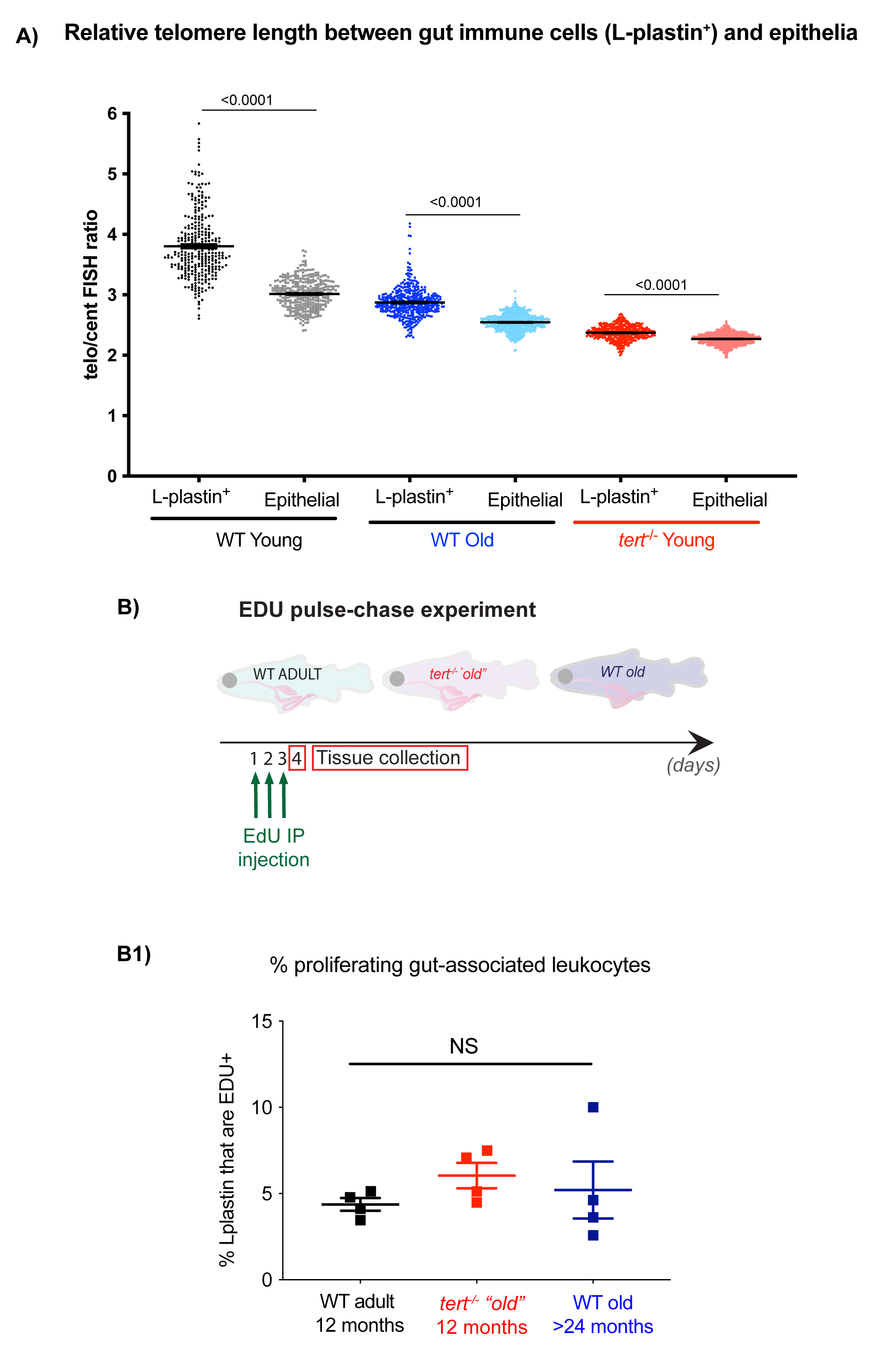

Supplement: Supplementary file 2 — Additional file 2: Supplementary Figure 2. Immune cells in the gut retain longer telomeres than epithelial cells, despite telomere shortening over time. A) Quantification of the relative telomere length of L-plastin+ cells, from gut paraffin sections, using combine immunostaining for anti-L-plastin and telomere in situ hybridization (Telo-FISH), together with the near-centromeric probe (Cent-FISH), as in Figure 1. Young animals are c.5 months old and old animals are >30-36 months old. B) EdU pulse-chase experiment schematics, where fish were injected with EdU by IP for 3 consecutive days to label all proliferating cells. Gut tissue was collected on day 4 (1-day post-chase) and B1) the % of Edu+ L-plastin+ cells was quantified and averaged per animal per genotype. [file 12979_2022_287_MOESM2_ESM.tif]
